# A wider and deeper peptide-binding groove for the class I molecules from B15 compared to B19 chickens correlates with relative resistance to Marek’s disease

**DOI:** 10.4049/jimmunol.2200211

**Published:** 2023-03-01

**Authors:** Lingxia Han, Shaolian Wu, Ting Zhang, Weiyu Peng, Min Zhao, Can Yue, Wanxin Wen, Wenbo Cai, Min Li, Hans-Joachim Wallny, David W. Avila, William Mwangi, Venugopal Nair, Nicola Ternette, Yaxin Guo, Yingze Zhao, Yan Chai, Jianxun Qi, Hao Liang, George F. Gao, Jim Kaufman, William J. Liu

**Affiliations:** * State Key Laboratory of Veterinary Biotechnology, Harbin Veterinary Research Institute, Chinese Academy of Agricultural Sciences, Harbin 150001, China; † National Poultry Laboratory Animal Resource Center, Harbin 150001, China; ‡ Heilongjiang Provincial Key Laboratory of Laboratory Animal and Comparative Medicine, Harbin 150069, China; § CAS Key Laboratory of Pathogen Microbiology and Immunology, Institute of Microbiology, Chinese Academy of Sciences, Beijing 100101, China; ¶ Biosafety Level-3 Laboratory, Life Sciences Institute & Collaborative Innovation Centre of Regenerative Medicine and Medical BioResource Development and Application, Guangxi Medical University, Nanning, Guangxi 530021, China; ‖ NHC Key Laboratory of Biosafety, National Institute for Viral Disease Control and Prevention, Chinese Center for Disease Control and Prevention, Beijing 100052, China; # Research Unit of Adaptive Evolution and Control of Emerging Viruses, Chinese Academy of Medical Sciences, Beijing 100052, China; ** Savaid Medical School, University of Chinese Academy of Sciences, Beijing 100049, China; †† The Basel Institute for Immunology, Basel CH4001, Switzerland; ‡‡ The Pirbright Institute, Pirbright GU24 0NF, United Kingdom; §§ Department of Zoology, University of Oxford, Oxford OX1 3SZ, United Kingdom; ¶¶ Nuffield Department of Medicine, University of Oxford, Headington OX37BN, United Kingdom; ‖ ‖ Department of Pathology, University of Cambridge, Cambridge CB2 1QP, United Kingdom; ## Department of Veterinary Science, University of Cambridge, Cambridge CB3 0ES, United Kingdom; *** Institute for Immunology and Infection Research, University of Edinburgh, Edinburgh EH9 3FL, United Kingdom

**Keywords:** disease susceptibility, peptide presentation, chicken MHC class I, Marek’s disease, BF2*1901, BF2*1501

## Abstract

The chicken major histocompatibility complex (MHC) is known to confer decisive resistance or susceptibility to various economically-important pathogens, including the iconic oncogenic herpesvirus that causes Marek’s disease (MD). Only one classical class I gene, BF2, is expressed at a high level in chickens, so it was relatively easy to discern a hierarchy from well-expressed thermostable fastidious specialist alleles to promiscuous generalist alleles that are less stable and expressed less on the cell surface. The class I molecule BF2*1901 is better expressed and more thermostable than the closely-related BF2*1501, but the peptide motif was not simpler as expected. Here, we confirm for newly-developed chicken lines that the chicken MHC haplotype B15 confers resistance to MD compared to B19. Using gas phase sequencing and immunopeptidomics, we find that BF2*1901 binds a greater variety of amino acids in some anchor positions than BF2*1501. However, by X-ray crystallography, we find that the peptide-binding groove of BF2*1901 is narrower and shallower. Though the self-peptides bound to BF2*1901 may appear more various than those of BF2*1501, the structures show that the wider and deeper peptide-binding groove of BF2*1501 allows stronger binding and thus more peptides overall, correlating with the expected hierarchies for expression level, thermostability and MD resistance. Our study provides a reasonable explanation for greater promiscuity for the BF2*1501 compared to BF2*1901, corresponding to the difference in resistance to MD.

## Introduction

The global pandemic of COVID-19 among humans caused by the coronavirus SARS-CoV-2 has emphasized the importance of understanding the mechanisms of resistance against viral pathogens (1). Compared to roughly 7 billion humans, there are estimated to be over 80 billion chickens alive each year, most of which are potentially subject to local epidemics by a variety of economically-important viral diseases (http://www.fao.org/faostat/en/#data/QL). The first coronavirus ever described causes infectious bronchitis in chickens and is still a major problem for commercial flocks (2, 3), but the iconic chicken pathogen is Marek’s disease virus (MDV), an oncogenic herpesvirus for which most commercial chickens are vaccinated and which still causes major economic losses due to changes in virulence and tropism (4–6). Much ongoing research is dedicated to determining the genetic loci responsible for resistance to Marek’s disease (7–9), but the BF-BL region within the B locus, which is clearly the functional equivalent of the major histocompatibility complex (MHC), has been known for decades to determine resistance and susceptibility (10–12).

In contrast to humans and other typical mammals, the chicken MHC is small and simple, and can determine striking resistance or susceptibility to a variety of economically-important infectious diseases (13). Compared to typical mammals which express multigene families of classical MHC class I molecules, in chickens only the BF2 molecule is well-expressed and is the major ligand for cytotoxic T lymphocytes, while the BF1 molecule acts as a ligand for natural killer (NK) cells and is relatively poorly expressed if at all (14). The presence of a dominantly-expressed classical class I molecule whose properties can determine the immune response has been suggested to be one reason why the chicken MHC has such strong genetic associations with infectious diseases (15), although other closely-linked genes may also be involved (16, 17).

The simplicity of the chicken MHC compared to typical mammals has allowed the discovery of some fundamental properties of classical MHC molecules, in particular the properties of class I molecules leading to the proposal of generalist and specialist alleles (18–20). In chickens, there is a clear hierarchy of class I alleles from so-called fastidious molecules that bind a narrow range of peptides, are relatively stable with the peptides naturally bound and have a relatively high cell surface expression compared to so-called promiscuous molecules that bind a wider variety of peptides, are overall less stable and have a lower expression at the cell surface (15, 18, 21–23). It has been relatively easy to understand the size of the peptide repertoire from the structures of the chicken class I molecules (15, 18, 22, 24–26), although peptide transport by TAP molecules and peptide editing by tapasin (TAPBP) may also contribute (23, 27, 28). The chicken MHC haplotypes with promiscuous class I alleles are generally associated with resistance to a variety of infectious viruses, including those responsible for Marek’s disease, infectious bronchitis, avian influenza and Rous sarcoma (19, 20)

A similar hierarchy of human classical class I alleles has been discerned (18–20, 29–31). For human class I alleles, the original observation was that fastidious class I molecules (so-called elite controller alleles) correlated with slow progression from HIV infectious to AIDS (19, 20, 29, 30), apparently due to binding special pathogen peptides that the virus cannot change for immune evasion without lowering viral fitness (32, 33). Based on assays of tapasin-dependence (34), these protective human alleles were correlated with dependence on the class I-bespoke chaperone tapasin (or TAPBP) in the peptide-loading complex (PLC) (18, 19, 29, 35). The results in chickens and humans led to the concept of generalist class I alleles that generally protect from many viral pathogens by binding a wide variety of peptides and specialist alleles that protect from particular pathogens by binding special peptides (18–20, 29). Most recently, it was found that promiscuous class I alleles in humans correlate with slow progression to AIDS if the elite controller alleles are removed from the analysis (35). The presence of fastidious class I alleles in chickens may also be explained by resistance to particular pathogens (20).

That chicken MHC haplotypes are in a hierarchy with respect to resistance to Marek’s disease is not in question, although the relative placement of particular haplotypes within that hierarchy has been debated (12, 36, 37). It is perhaps a surprise that any consensus could have arisen, given the differences between experiments in the relative virulence of different MHC strains, the route of infection, the measurement of disease, the chicken lines with different genetic backgrounds and the differences even within MHC haplotypes. For example, there are clearly two kinds of B15 haplotypes, those that have a functional BF1 gene and those that do not (15, 38, 39), and the relative resistance to MDV conferred by these haplotypes has not been examined. Moreover, there is evidence the BG1 gene within the chicken MHC can contribute to resistance to virally-induced tumours (16), with many of the infection experiments carried out before the BG1 gene was even discovered (40–42).

There seems to be little disagreement that the B19 haplotype confers the most susceptibility, and most experiments with B15 haplotypes show that it confers susceptibility but less than B19 (12, 36, 37). In agreement, the expression level and thermostability are higher for class I molecules on erythrocytes and splenocytes for B19 than B15 (19, 21, 23). However, it has not been obvious from published data on peptide motifs whether BF2 molecules from B19 are more fastidious than B15, nor has there been a structure for the BF2 molecule from a B19 haplotype. Using the established lines (43), we report the viral levels from a B15 and a B19 chicken line, describe the peptide motifs of the two haplotypes in much more detail than previously, determine structures for BF2*1901 with two peptides, and compare both BF2*1501 and BF2*1901 with the same peptide as well as with several other peptides, including one B15 structure recently published (24). From these analyses, we confirm that viremia for MDV is higher in the B19 than the B15 chicken lines, and find a structural explanation for greater promiscuity for the BF2 molecule from B15 compared to B19, correlating with the facts that BF2*1901 has higher surface expression, greater stability with peptides *in vivo* and confers susceptibility to Marek’s disease.

## Materials and Methods

### Animals

The BWEL chicken line, originating from Beijing white chickens which descend from Chinese native and White Leghorn chickens, is an important genetic resource of the Chinese State Resource Center of Poultry Laboratory Animals, affiliated with the Harbin Veterinary Research Institute (HVRI) of the Chinese Academy of Agricultural Sciences (CAAS). We successfully established six homozygous MHC-B haplotype populations from BWEL chickens using microsatellite marker technology, which were correlated with serological types and with gene sequences, including the chicken lines BW/G 5 and 7 containing B15 and B19 MHC haplotypes, respectively (43). The chicken populations were maintained under specific pathogen-free (SPF) situations in positive pressure isolators with high efficiency particulate air (HEPA) filters throughout life and have been free from 19 avian diseases, all of which conform to the request stipulated by national standard of GB 17999.1-2008 *SPF chicken-Microbiological surveillance-Part 1: General rules for the microbiological surveillance for SPF chicken* for 15 generations. The environment index of the breed facility conforms to the standard of GB 14925-2010, with ^60^Co-sterilized feed and acidified drinking water; the laboratory animal production license issued by local government is SCXK (HEI) 2006-009. The research was approved by Committee on the Ethics of Animal Experiments from HVRI.

### Expression levels of cytokine RNA

Four duplex TaqMan probe real-time fluorescence quantitative RT-PCR (dqRT-PCR) protocols for chicken IFN-γ, IL-18, IL-10 and IL-4 were established as previously described (44–46). Primers and probes were synthesized by Shanghai Sangong Biotechnology Co., LTD. Venous blood was collected in heparin as anti-coagulant from one B15 and one B19 bird followed by isolation of peripheral blood lymphocytes (PBL). Total RNA was extracted from PBL using the RNAisoTM Plus kit (TaKaRa, Dalian, China). The RT reaction was performed using reverse transcriptase M-MLV kit (TaKaRa). Standard curves for dqRT-PCR were carried out using pMD-chIFN-γ, pMD-chIL-18, pMD-chIL-10 and pMD-chIL4 recombinant plasmids constructed with the T-A vector of pMD18-T grown up in *E. coli* TG1.

### Infection and measurement of viral loads

The very virulent MDV strain MD5 was expanded by infection of pullets. Fresh or rejuvenated Md5-infected chicken peripheral blood was diluted by 50 times with DMEM, and 1-day-old B19 and B15 chickens (each n=9) were inoculated intraperitoneally (i.p.) with 500 μL each, a dose found previously to show differences in susceptibility (47, 48). Control animals (n=5 for each line) were inoculated with 500 μL DMEM. The feather pulps of 3 chickens in each group were collected randomly at 4, 7, 9, and 12 dpi. Virus copy number was detected by dqPCR using the MDV *meq* gene to detect the virus load, and with the chicken egg iron transfer protein gene (ovo) as an internal reference. We averaged two PCR tests and subtracted the results of the mock-infected chicken samples for the viral titer calculation.

### Sequencing of peptides bound to class I molecules

As described in detail (15, 18), monoclonal antibodies to chicken class I heavy chain (F21-2) and to chicken β_2_-microglobulin (F21-21) were used to isolate class I molecules from cells lysed in detergent: F21-21 once with H-B19 blood, and F21-2 once and F21-21 once with H-B19 spleen cells at the Basel Institute for Immunology, F21-21 once with P2a spleen cells at the Institute for Animal Health, and F21-2 once with the B19 cell line MDCC-265L at the Pirbright Institute. The peptides from the *ex vivo* cells were separated by reverse-phase HPLC using a Pharmacia SMART system, with sequencing of individual peptide peaks or of whole peptide pools using an Applied Biosystems 475A gas phase sequencing. The peptides from the cell line were analyzed by LC-MS/MS using the Q-Exactive (Thermo Scientific) and TripleTOF 5600 (AB Sciex) systems.

### Peptide synthesis and preparation of expression constructs

The extracellular region (corresponding to amino acids 1–270) of BF2*1901 (GenBank: Z54317.1, https://www.ncbi.nlm.nih.gov/nuccore/Z54317.1) was synthesized (Genewiz Inc, Beijing), cloned into a pET21a vector (Novagen) and transformed into *E. coli* strain BL21(DE3). The expression plasmid for chicken β_2_m (chβ_2_m) (expressing residues 1-98) was constructed previously in our laboratory (26). Potential chicken MHC I BF2*1901-binding peptides (Table S1, ref. 22) were synthesized and purified by reverse-phase high-performance liquid chromatography (HPLC) (SciLight Biotechnology, Beijing). The peptide purity was determined to be >95% by analytical HPLC and mass spectrometry. The peptides were stored at -80°C as freeze-dried powders and were dissolved in dimethyl sulfoxide (DMSO) before use (49).

### Refolding and purification of BF2*1901 and BF2*1501

Dilution-renaturation and purification of class I molecules assembled with peptides were performed as described previously (50). Firstly, 1 mL of dissolved denatured chβ_2_m inclusion bodies was dropped slowly to 500 mL refolding buffer (100 mM Tris-HCl pH 8.0, 2 mM EDTA, 400 mM L-Arg, 0.5 mM oxidized glutathione, 5 mM reduced glutathione) and incubated at 4°C for 0.5 h. Subsequently, 5 mg of peptide dissolved in DMSO were added to the solution. Half an hour later, 3 mL denatured BF2*1901 heavy chain inclusion bodies were added to the solution drop by drop. After incubation for 8 h, the soluble portion was concentrated and purified by chromatography on a Superdex 200 16/60 HiLoad (GE Healthcare) size-exclusion column.

### X-ray crystallography, structure determination, and refinement

Crystallization was performed using the sitting drop vapor diffusion technique. BF2*1901/RY8 crystals were observed in 0.15 M KBr and 30% w/v polyethylene glycol monomethyl ether 2000 at a protein concentration of 13.5 mg/mL. Single crystals of BF2*1901/IL9 were grown in 0.1 M BIS-TRIS pH 6.5 and 28% w/v polyethylene glycol monomethyl ether 2000 at a protein concentration of 12.5 mg/mL. Diffraction data for both crystals were collected at 100 K at the SSRF BEAMLINE BL17U, Shanghai, China. The collected intensities were subsequently processed and scaled using the DENZO program and the HKL2000 software package (HKL Research) (51). The structure of BF2*1901 was determined by molecular replacement using BF2*1201 (Protein Data Bank [PDB] code 5YMW) as a search model in the Crystallography & NMR System (CNS) (52) and COOT (53), refined with REFMAC5 (54) and PHENIX (55), and assessed with PROCHECK (56) (Table 1). Structure-related figures were generated using PyMOL (http://www.pymol.org/). The sequence alignment was generated with Clustal X (57) and ESPript (58).

### Determination of thermostability using circular dichroism (CD) spectroscopy

To compare the thermostability of BF2*1901 and BF2*1501 bound to a similar peptide, we used CD spectroscopy as previously described (49). All complexes were prepared as described above and diluted to 0.2 mg/mL in 20 mM Tris−HCl (pH 8.0) and 50mM NaCl. Thermal denaturation curves were determined by monitoring the CD value at 218 nm using a 1-mm optical path-length cell as the temperature was raised from 20 to 100 °C at a rate of 1 °C/min. The temperature of the sample solution was directly measured with a thermistor. The fraction of unfolded protein was calculated from the mean residue ellipticity (θ) by the standard method. The unfolded fraction (%) is expressed as (θ - θ_N_) / (θ_U_ - θ_N_), where θ_N_ and θ_U_ are the mean residue ellipticity values in the fully folded and fully unfolded states, respectively. The midpoint transition temperature (*T*_m_) indicates by the temperature when 50% of the protein unfolded determined by curves using the Origin 8.0 program (OriginLab).

### Accession numbers

Protein Data Bank (http://www.rcsb.org) accession codes are 7WBG for BF2*1901/RY8 and 7WBI for BF2*1901/IL9.

## Results

### The expression of three cytokines is higher in B19 than in B15 naïve SPF chickens

To determine the baseline differences of T cell responses for the two haplotypes B15 and B19, we selected cytokines from CD8^+^ T cells which in chickens recognize BF2 molecules. The transcription levels of IFN-γ, IL-18, and IL-10 in the healthy chickens were investigated by the dqRT-PCR. The amplification curves in each reaction were standard “S” type, and the amplification efficiencies of the target and reference genes were similar, and showed good linear relationships. Based on the quality-controlled dqRT-PCR, mRNA expression was quantified to compare the natural cellular immunological level between the SPF B15 and B19 chicken lines.

**Fig. 1:**
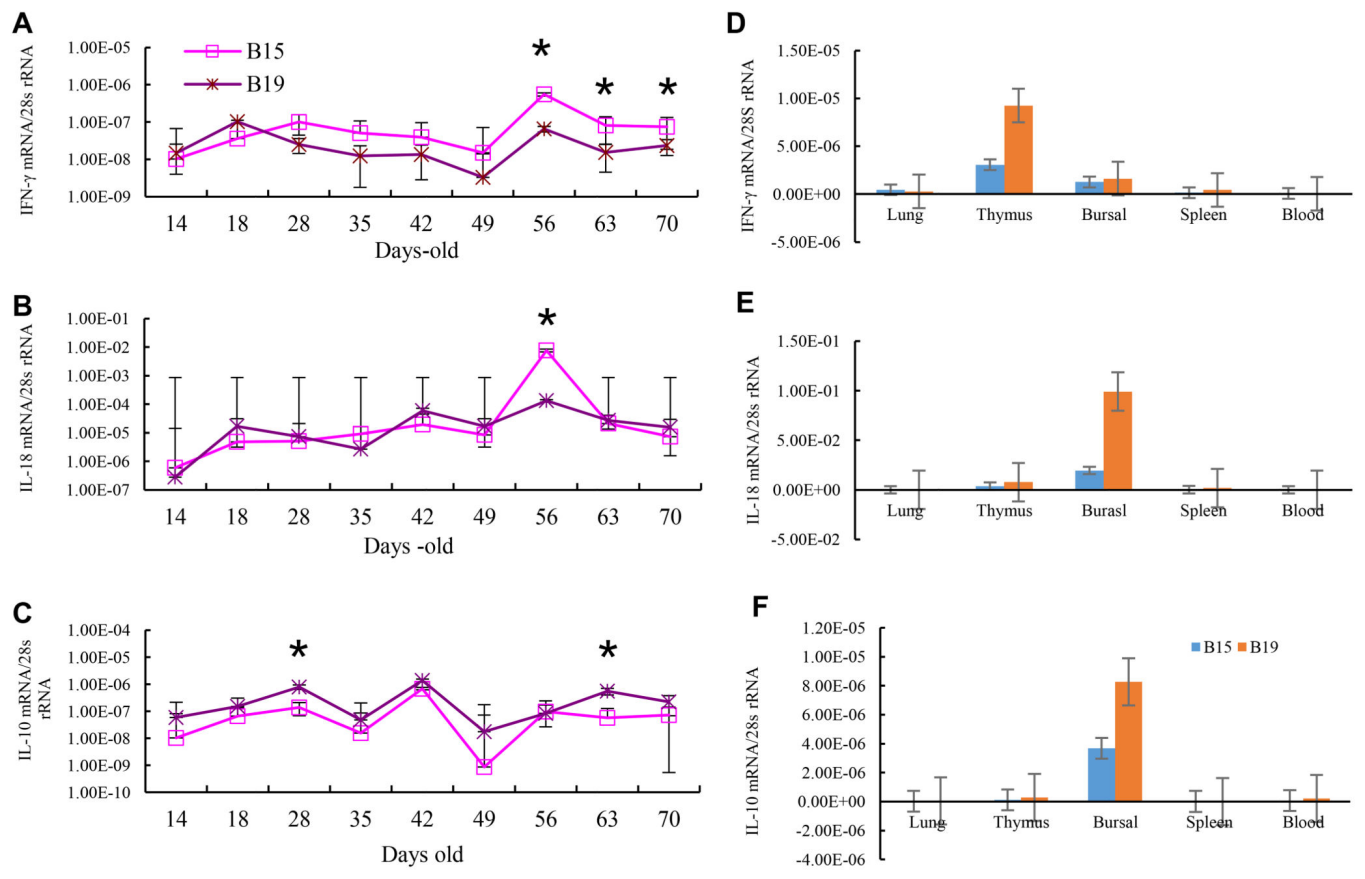
The relative background cytokine expression in B15 and B19 SPF chickens and in different tissues of B15 and B19 at 70d-old. The relative expression levels of IFN-γ (A), IL-18 (B), and IL-10 (C) in peripheral blood were detected with dqRT-PCR method. The data came from 11 healthy B15 chickens and 6 of B19 chickens at different days old. The relative expression levels of IFN-γ (D), IL-18 (E), and IL-10 (F) in lung with respiratory tract, thymus, bursa of Fabricius, spleen and peripheral blood were detected with dqRT-PCR method. The data came from each 3 of 70 days-old B15 and B19 chickens. The experiments were independently performed twice. *, P<0.05.

B19 chickens have the less IFN-γ levels in PBLs from 28 through 70 days-old than B15 chickens with significant differences on day 56, 63 and 70. Meanwhile, B19 chickens have higher IL-10 in PBLs than B15 chickens with significant differences on day 28 and 63. No difference was observed for IL-18 except at 56 days-old (Fig. 1A-C).

Secondly, the relative expression levels of IFN-γ, IL-18, and IL-10 in lung and respiratory tract, thymus, bursa of Fabricius, spleen and peripheral blood were detected at 70 d-old, since generally the immune organs of chicken mature by 2 months of age. All three cytokines were expressed in the primary lymphoid organs of thymus and bursal, but only at a low level in respiratory system, spleen and PBLs. The expression levels differed among the tissues and cytokines, with IL-10 and IL-18 transcribed mainly in the bursa and IFN-γ mainly in thymus. Significantly, the three cytokines were expressed more in the corresponding organs of B19 chickens compared to B15 chickens (Fig. 1D-F).

### Difference in disease susceptibility between B19 and B15 chickens infected by MDV Md5

**Fig 2:**
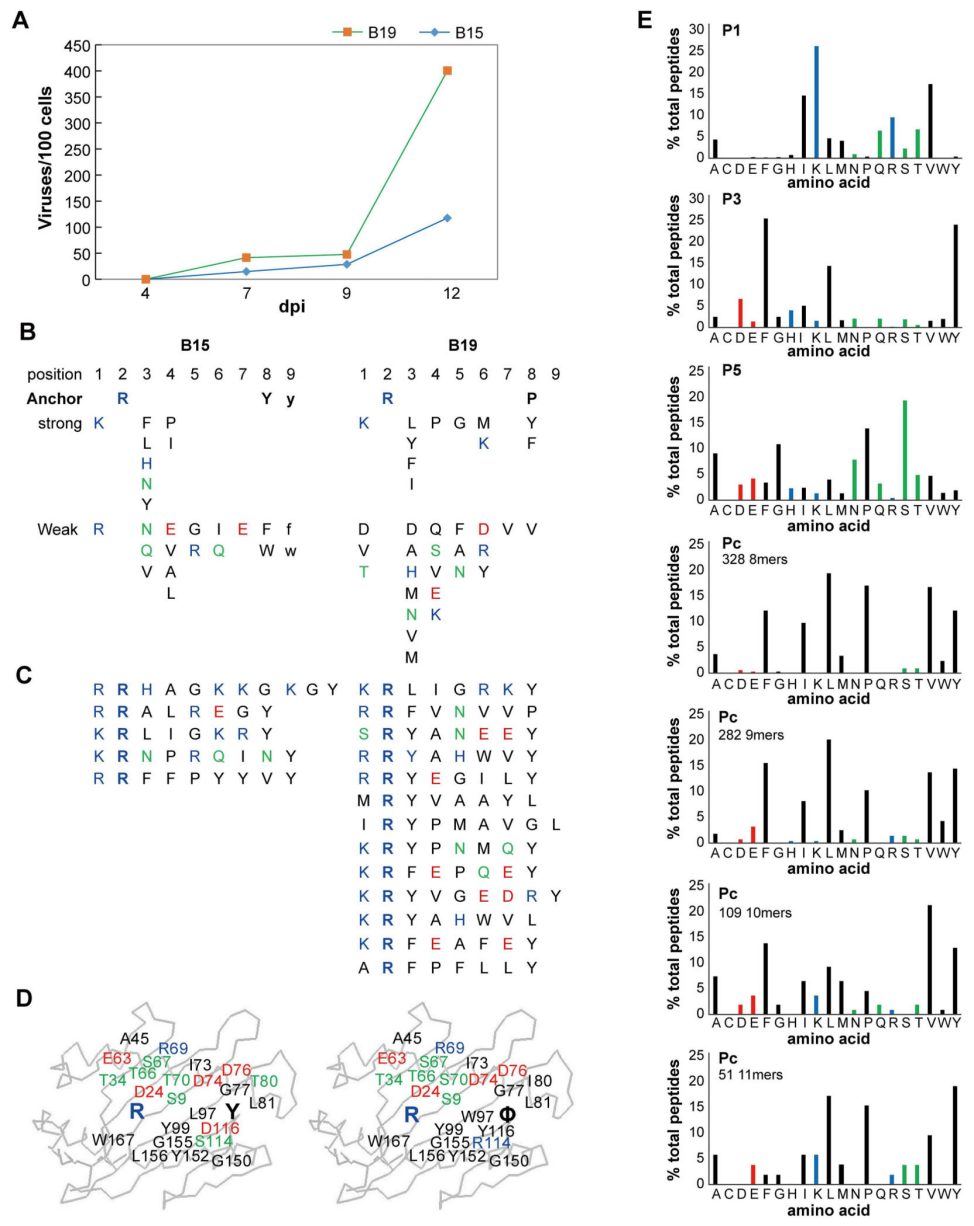
Susceptibility of B15 and B19 SPF chickens to MDV Md5 and peptide preference of BF2*1501 and BF2*1901. A, The dynamic viral load of MDV Md5 in B19 and B15 haplotype chickens at 4, 7, 9, and 12 dpi. The data came from each 3 B15 and B19 chickens for each time spot. We averaged two PCR tests and subtracted the results of the mock-infected chicken samples for the viral titer calculation. B-D. Self-peptides bound to BF2*1501 and BF2*1901 as assessed by gas phase sequencing. B, Sequences of peptides bound to class I molecules isolated from red blood cells determined from peptide pools showing anchor, strong and weak signals. C, Sequences of individual peptides separated by HPLC. D, Peptide anchor residues in large letters superimposed on a wire model of class I α1 and α2 domains with those residues that are both polymorphic and potentially peptide contacts indicated as smaller letters; numbering based on HLA-A2 sequence. Single letter code for amino acids (with Φ for hydrophobic); basic residues in blue, acidic residues in red, polar residues in green, hydrophobic residues in black. Results and analysis for B15 adapted from previous study (15). E, Analysis of peptides from B19 cells as assessed by immunopeptidomics. Bar graphs showing the frequency (y-axis) of each natural amino acid (single letter code: basic residues in blue, acidic residues in red, polar residues in green, hydrophobic residues in black; x-axis) for peptides eluted from an MDV-transformed cell line MDCC-265L which have Arg at P2 (thus likely to be BF2*1901), for peptide positions P1, P3 and P5, and for the C-terminal amino acid (called Pc or Pω) separated by peptide length and with the number of each length indicated. Monoclonal antibodies to chicken class I heavy chain (F21-2) and to chicken β_2_m (F21-21) were used to isolate class I molecules from cells lysed in detergent: F21-21 once with H-B19 blood, and F21-2 once and F21-21 once with H-B19 spleen cells at the Basel Institute for Immunology, F21-21 once with P2a spleen cells at the Institute for Animal Health, and F21-2 once with the B19 cell line MDCC-265L at the Pirbright Institute. Original data from experiment (18) shown in Supplemental dataset 1.

One-day old chickens were inoculated i.p. with the very virulent Md5 strain of MDV. At 4, 7, and 9 dpi, the number of virus copies within the feather pulps of B19 and B15 chickens remained quite similar. However, at 12 dpi, the virus copy numbers in B19 chickens were much higher than B15 chickens (Fig. 2A). At 20 dpi, two chickens died in the B19 group and one chicken died in the B15 challenge group. The spleen and kidney of the dead chickens infected with B19 were enlarged, the thymus glands were atrophied, and the liver was congested with the surface color darkened. The livers were atrophied and the kidneys were enlarged in the B15 chickens that died, but the surface color of the livers was lighter.

### Peptides and peptide motifs from class I molecules of B15 and B19 chickens

As mentioned in the introduction, isolation of class I molecules from chicken blood and spleen cells followed by HPLC and gas phase sequencing of single peptides and peptide pools provided the first glimpses into how chicken class I molecules bind peptides. This first description involved what now might be called fastidious molecules with multiple simple anchor residues, and showed that the class I molecules from the B15 and B19 haplotypes had very similar motifs (21), with an Arg at peptide position 2 (R2) for both, a Tyr at Pc (also called PΩ, in this case P8 or P9) for B15, and a few hydrophobic amino acids (including Leu, Phe, Pro and Tyr) at Pc (P8) for B19. We now know that chickens typically have a BF2 that is the dominantly-expressed class I gene: B19 has a poorly-expressed BF1 gene and most B15 haplotypes have no functional BF1 gene (15, 59), so these gas phase sequencing results reflect the peptides from the BF2 molecule. The B15 peptides and motifs were described in detail (15, 22), but the detailed B19 results are only presented now (Fig. 2B, C). A pool sequence and 13 individual peptides confirm the initial points: both 8mers and 9mers are found for B15, but B19 has mostly 8mers; both B15 and B19 have Arg for P2; B15 is mostly Tyr (with some Phe and other hydrophobic amino acids) at Pc, but B19 has Tyr, Pro, Leu (and some Phe) at Pc. In addition, B15 has entirely basic residues Arg and Lys at P1, while B19 has mostly Lys but some Arg and some hydrophobic residues. Finally, the two motifs fit well with wire models of the class I molecules (as described for B15, ref. 15) (Fig. 2D), predicting that basic residues at P1 and Arg at P2 interact with the acidic residues E63 and D24 in both molecules, and with Tyr at Pc sitting in a hydrophobic pocket with the hydroxyl interacting with D116 of B15, but the hydrophobic amino acids at Pc interacting with a more hydrophobic pocket in B19.

More recently, isolation of class I molecules from a B19 cell line followed by HPLC and mass spectrometric analysis of single peptides (LC-MS/MS, or immunopeptidomics) was performed (Supplemental dataset 1) (18), which confirmed and extended the previous results. As this cell line expressed BF1 molecules at a higher level than is found on normal cells, an analysis of the peptides with Arg at P2 (almost certainly from the BF2 molecule) was performed (Fig. 2E). Of the 896 peptides, amino acids at P1 were over 25% Lys, 17% Val and 15% Ile, with lesser amounts of Arg, Gln and Thr and then Ala, Leu, Met and Ser. Over 50% of all peptides had either Phe or Tyr at P3, with around 15% Leu and lesser amounts of other amino acids. At P5, 19% Ser, 14% Pro, 11% Gly, 9% Ala and other amino acids at lower amounts were found. Around 82% of amino acids at Pc were Phe, Ile, Leu, Pro, Val or Tyr, although which predominated depended on the length of peptide, for which there were 328 8mers, 282 9mers, 109 10mers and 51 11mers (totaling 770 of the 896 peptides, with nearly all of the rest being longer).

### The structural overview of BF2*1901 is similar to BF2*1501

**Fig 3:**
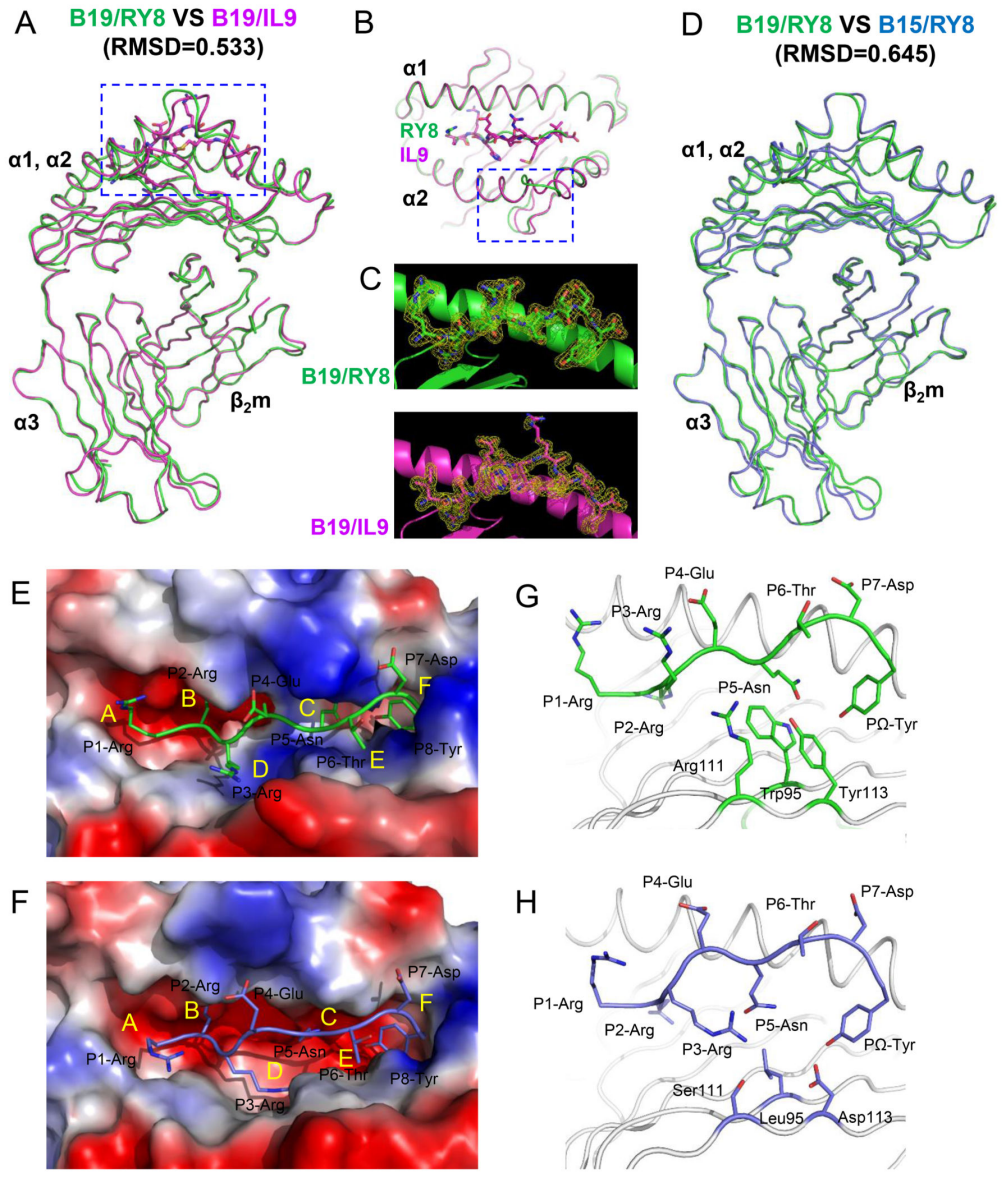
Structural overview of BF2*1901 and the shallow and narrow peptide binding groove compared to BF2*1501. A, Superimposed overall structures of BF2*1901 complexed to MDV peptide RY8 (green), and influenza H5N1 virus M1 peptide IL9 (purple). The RMSDs of the MHC monomers were determined to be 0.533 Å for B19/RY8 versus B19/IL9. B, The alignment of α1α2 domain of B19/RY8 and B19/IL9 indicates a conformational difference in the α2 helices of the two structures. C, The peptide RY8 (green) and IL9 (purple) in the structures of BF2*1901 are presented with the 2Fo-Fc electron density maps at the 1.0 σ contour level. D, Superimposed overall structures of B19/RY8 (green) and BF2*1501 complexed to the same MDV peptide RY8 (PDB: 6LHH, blue). The RMSDs of MHC monomers were determined to be 0.645 Å for B19/RY8 versus B15/RY8. E, The electrostatic plot shows the peptide binding groove of BF2*1901 complexed to MDV peptide RY8 (green). F, The electrostatic plot shows the peptide binding groove of BF2*1501 complexed to MDV peptide RY8 (PDB: 6LHH, blue). G, The peptide RY8 in BF2*1901 is shown in green cartoon with three large B19-specific residues at the bottom of the groove (Trp95, Arg111, Tyr113) shown in green sticks. H, The peptide RY8 in BF2*1501 (PDB: 6LHH) is shown in blue cartoon, with three B15-specific residues at the bottom of the groove (Leu95, Ser111, Asp113) with short side chains shown in blue sticks.

On the basis of the motifs determined above, peptides from MDV that might bind different chicken MHC molecules were predicted (22) and the peptide RY8 (RRRENTDY) was selected because it was predicted to bind both BF2*1501 and BF2*1901, and shown to bind BF2*1501 (60). In addition, peptides from avian influenza viruses were predicted (Table 1, Table S1) and the influenza H5N1 virus M1 peptide IL9 (IRHENRMVL) was found to bind BF2*1901. The structure of chicken class I molecule BF2*1901 complexed with MDV peptide RY8 was determined to resolution of 2.0 Å with two molecules in one asymmetric unit, while the structure of BF2*1901 with influenza virus peptide IL9 was determined at 2.0 Å with one molecule in an asymmetric unit. The overall structure of BF2*1901 retains the common characteristics of MHC class I molecules from other vertebrates including chickens: the extracellular region of the BF2*1901 heavy chain folds into three different domains (Fig. 3A); the α1 and α2 domains form a typical MHC I peptide binding groove (PBG), which contains two α-helices and eight β-strands; the RY8 or IL9 peptide lies along the PBG, as shown by well-determined electron density maps (Fig. 3B, C). The α3 domain of BF2*1901 and β_2_m are typical immunoglobulin superfamily domains and underpin the α1 and α2 domains. The Cα atom superposition of BF2*1901/RY8 onto BF2*1901/IL9 generated a root mean square deviation (RMSD) of 0.533 Å. The superposition of these two structures showed that the most distinct portion of the two molecules is located in the middle of the α2 helix, covering residues Glu145 to Tyr149 (Fig. 3B). For BF2*1901/IL9, the loop at the middle of α2 helix is closer to the C-terminus of the peptide, compared to the structure of BF2*1901/RY8.

The overall structures of BF2*1901 are extremely similar to those of BF2*1501 (Fig. 3D). The Cα atom superposition of BF2*1901/RY8 onto the previously determined structure of BF2*1501 complexed to the same peptide RY8 generated an RMSD of 0.645 Å. Moreover, the identity of the amino acid sequences of BF2*1901 and BF2*1501 is 97.04% (Fig. S1). As expected, only the two polymorphic residues S69T and I79T (for BF2*1901 versus BF2*1501) are located in the α1 and α2 helices. The structural comparison highlights the altered solvent exposure of residue I79T, which may have important role in the distinct MHC restrictions for T-cell receptor (TCR) recognition. As for the conformation of the main chain, BF2*1901/RY8 has a similar conformation as BF2*1501 at the middle of the α2 helix, while BF2*1901/IL9 has a conformational shift at this place (Fig. 3B,D).

### The shallow and narrow peptide binding groove of BF2*1901

Like most mammalian classical class I molecules as well as BF2*1501, BF2*1901 has obvious pockets A–F (Fig.3E). However, only the pockets A and B of BF2*1901 are very similar to BF2*1501, while the C, D, E and F pockets in the PBG of BF2*1901 possess their own allele-specific features. Pockets A of BF2*1501 and BF2*1901 present a large and open space with a relative negative charge to accommodate the P1 residue at the N-terminus of peptide. Furthermore, the B pockets for both BF2*1501 and BF2*1901 are very deep and negatively-charged. The conserved salt bridges between the P2-Arg of the peptides and residues Asp24, Thr34, and Glu62 of the main chains of both BF2*1501 and BF2*1901 can be observed (Fig. S2A, B).

In contrast, the major distinct portions of the PBG of BF2*1501 and BF2*1901 locate to the C, D, and E pockets in the center of the groove. Compared to BF2*1501, BF2*1901 has a much narrower and shallower groove (Fig. 3E, G). The main-chain atoms of the α1/α2 platform of BF2*1901 and BF2*1501 are nearly superimposable (Fig. 3D), so that the differences in groove width are due entirely to the different side chains of amino acids pointing into the groove. In particular, the large residues Trp95, Arg111 and Tyr113 from the β-strands on the bottom of PBG of BF2*1901 are replaced by the much smaller Leu95, Ser111 and Asp113 in BF2*1501 (Fig. 3F, H). The large overhanging residues with bulky side chains occupy most of the space in the C, D, and E pockets in PBG of BF2*1901. The distances from the bottom of the PBG to the bound peptide (represented by the upper atom of the side chain of Trp95, Arg111 and Tyr113 to the corresponding Cα-atom of P4, P5, and P6 residues of the peptides) are 4.76 Å, 4.89 Å and 6.59 Å, compared to the longer distances in BF2*1501, i.e. 7.00 Å, 10.16 Å, and 9.16 Å. Thus, the deep and wide middle portion of PBG of BF2*1501 allows the groove to accept peptides with promiscuous secondary anchor residues in the middle and to adopt various conformations.

### The tight but flexible P1 anchor of BF2*1501 compared to BF2*1901

**Fig 4:**
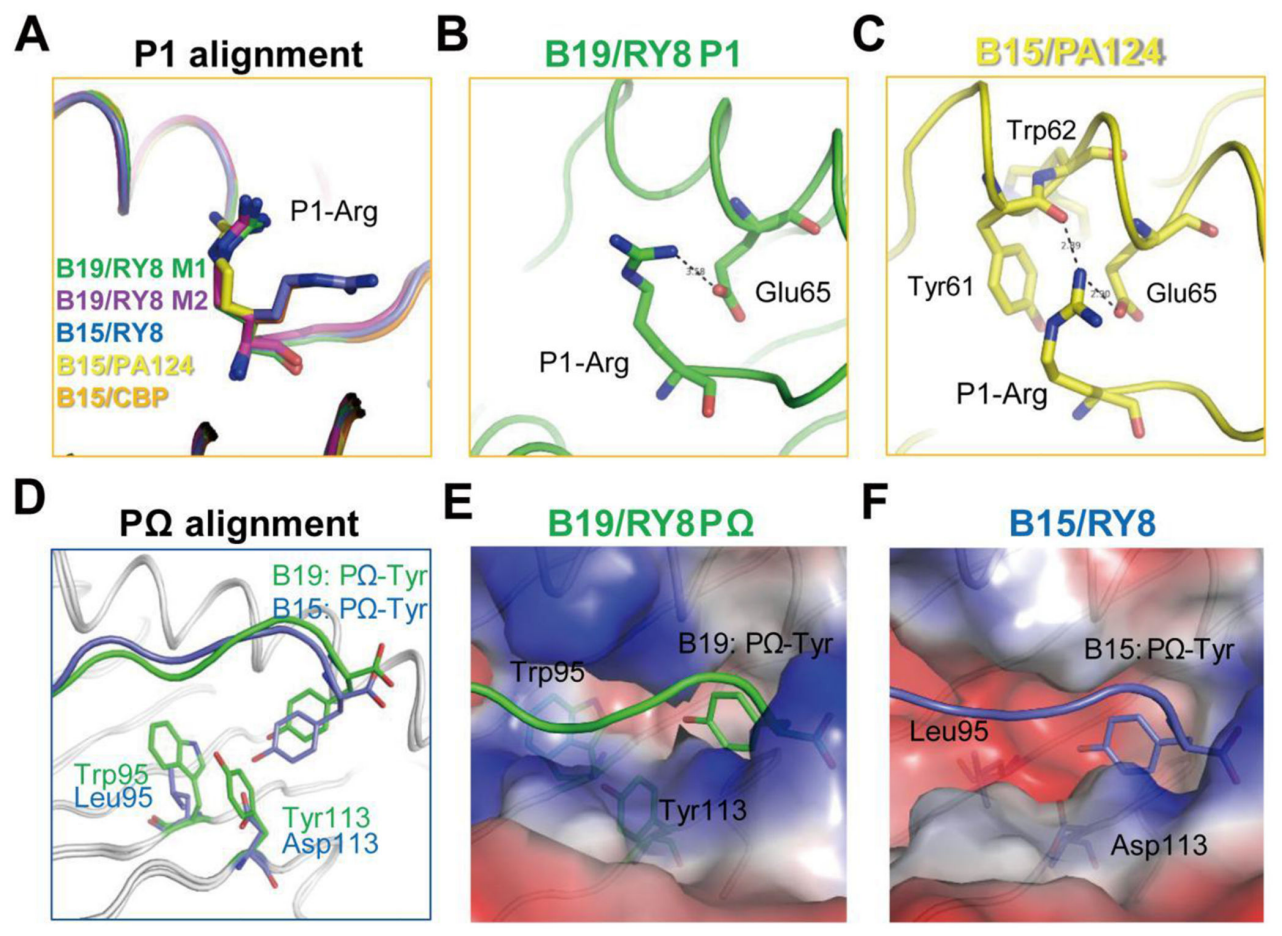
The different features of A and F pockets of BF2*1901 compared to BF2*1501. A, The superposing between A pockets of the two molecules M1 (green) and M2 (purple) of the asymmetrical unit of B19/RY8 structure, and also BF2*1501 complexed to MDV peptide RY8 (PDB: 6LHH, blue), flu peptide PA124 (PDB: 6IRL, yellow) and chicken calcium-binding protein peptide CBP (PDB: 6KX9, orange). The superposition clearly shows the similar conformation of P1-Arg in BF2*1901 M1 and M2, but the P1-Arg of B15/PA124 is closer to the α1 helix, while the P1-Arg of peptides RY8 and CBP are closer to the peptide itself. B, The weak hydrogen bond between P1-Arg of peptide RY8 (green) and Glu65 (green) in B19/RY8 M1. No interaction between them is observed in B19/RY8 M2 (not shown). C, The closer binding between P1-Arg in B15/PA124 (yellow) and the residues Tyr61 and Glu65 in α1 helix of BF2*1501. D, Superposition of B19/RY8 and B15/RY8 according to the α-C of α1α2 domains, clearly showing the higher position of PΩ-Tyr of peptide RY8 in B19/RY8 structure, compared to the PΩ-Tyr of peptide RY8 in B15/RY8 structure. E and F, The electrostatic plot shows the narrow and shallow F pocket of BF2*1901 (E, peptide RY8 in green sticks) compared to the BF2*1501 (PDB: 6LHH) (F, peptide RY8 in blue sticks).

In the structure of BF2*1501 and BF2*1901, the conserved residue Glu65 enables the A pocket to be relatively negatively-charged, as it is in HLA-B27 (61). Thus, the peptides with positive charged P1 residues are preferred by both BF2*1501 and BF2*1901 (15, 21). However, the detailed superposition of the two chicken class I molecules shows different modes of P1 anchoring. We superposed A pockets of the BF2*1901/RY8 and all the available structures of BF2*1501 complexed to peptides with positive P1 anchors (Fig. 4A). The superposition clearly shows the similar conformation of P1-Arg in the two molecules M1 and M2 of the asymmetrical unit of B19/RY8 structure (Fig. 4A), but the P1-Arg of flu peptide PA124 presented by BF2*1501 is closer to the α1 helix (Fig. 4A) while the P1-Arg of peptides RY8 and chicken calcium-binding protein peptide CBP in BF2*1501 structure is closer to the peptide itself (Fig. S2C). Moreover, in the M1 of BF2*1901/RY8, the hydrogen bond between P1-Arg of peptide RY8 and the Glu65 of BF2*1901 is 3.58 Å (Fig. 4B), while no interaction between them is observed in B19/RY8 M2 (not shown). In contrast, closer and stronger binding with two hydrogen bonds between P1-Arg in B15/PA124 and the residues Tyr61 (2.89 Å) and Glu65 (2.90 Å) in α1 helix of B15 can be observed (Fig. 4C).

### The narrow and shallow F pocket of BF2*1901

Then we focused on the PΩ anchors, for which BF2*1501-binding peptides strongly prefer Tyr, but BF2*1901-binding peptides have a variety of hydrophobic anchor residues. When we superposed the structures of B19/RY8 and B15/RY8 according to the Cα of α1/α2 domains, we found that the position of PΩ-Tyr of peptide RY8 in B19/RY8 structure is higher compared to RY8 in B15/RY8 structure (Fig. 4D). The solvent-accessible surface area of PΩ-Tyr that is buried upon interface formation with the pocket F in BF2*1901 (246.94 Å^2^ for molecule 1 and 219.72 Å^2^ for molecule 2 in the asymmetric unit cell) is smaller than PΩ-Tyr in BF2*1501 structure (260.38 Å^2^). The detailed analysis of BF2*1901 shows the narrow and shallow F pocket occupied by the residues Trp95 and Tyr113 with large side chains (Fig. 4E). Thus, the B19-specific residues Trp95 and Tyr113 act like two bricks to bolster up the PΩ-Tyr of peptide RY8 in B19. In contrast, residues Leu95 and Asp113 in the F pocket of B15/RY8 make room for the deep location of PΩ-Tyr of peptide RY8 in B15 (Fig. 4F). We calculated the volumes of the F pockets, and found that BF2*1901 (93.27 Å^3^) truly has a smaller F pocket compared to BF2*1501 (108.84 Å^3^). Furthermore, the relatively smaller F pocket of BF2*1901 can accommodate the peptide IL9 with PΩ-Leu (Fig. S2D), which is never found for BF2*1501 (Fig. 2B, E).

### The flexible but tight binding of P3 side chain of BF2*1501 compared to BF2*1901

**Fig 5:**
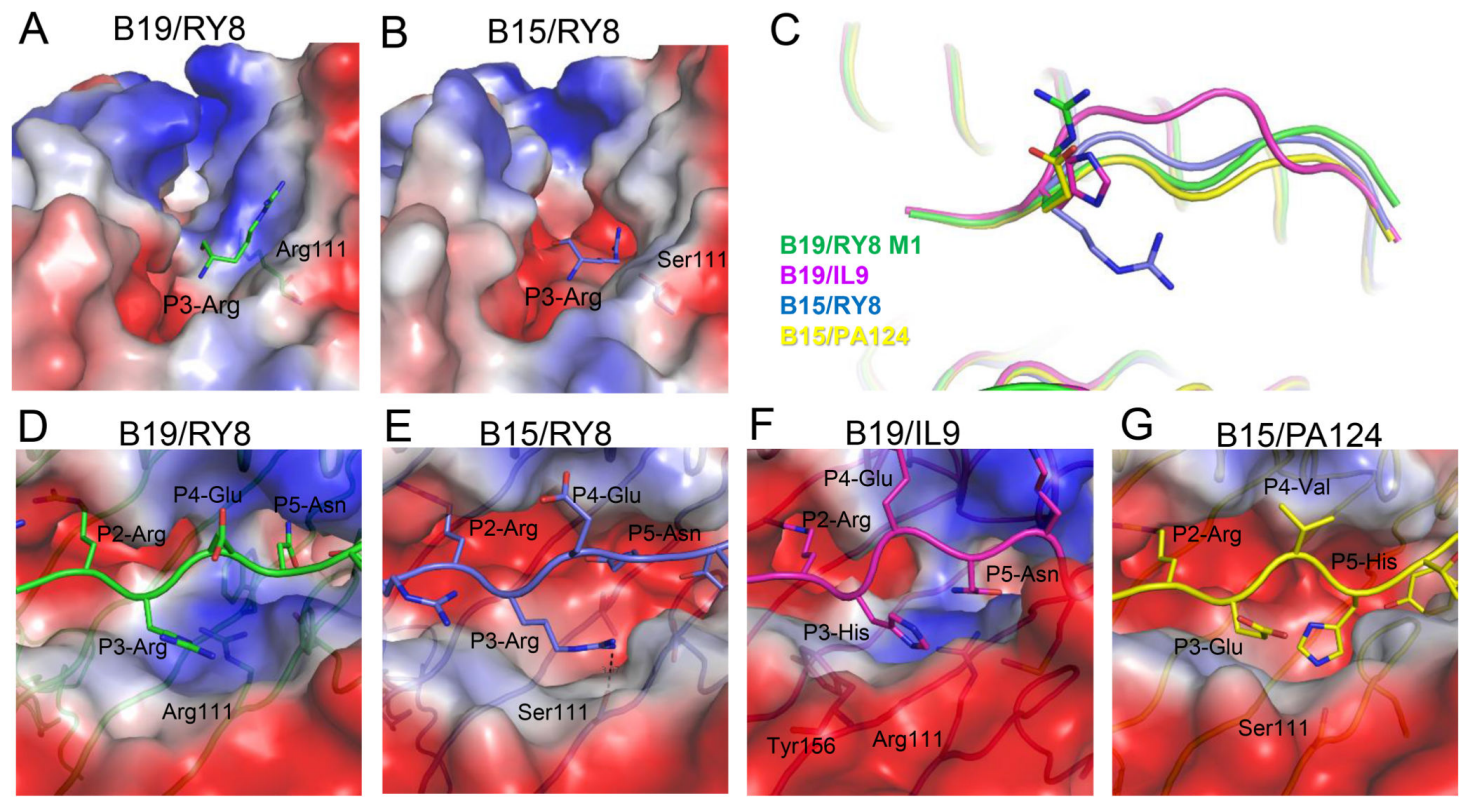
The flexible but tight binding of P3 anchor of BF2*1501 compared to BF2*1901. A, The P3-Arg anchor protrudes its side chain out of the shallow and narrow D pocket of B19/RY8 groove, which is occupied by the large positively-charged residue Arg111. B, The P3-Arg anchor puts its side chain into the D pocket of B15/RY8 groove (PDB: 6LHH), which is occupied by the small residue Ser111. C, Superposition of B19/RY8 (green), B19/IL9 (purple), B15/RY8 (PDB: 6LHH, blue) and B15/PA124 (PDB: 6IRL, yellow), clearly showing two different conformations of P3 anchors of peptides presented by BF2*1501, i.e. P3-Arg in RY8 and P3-Glu in PA124. D, The smaller D pocket of B19/RY8. E, The larger D pocket of B15/RY8 and the hydrogen bond between P3-Arg and Ser111 of BF2*1501. F, The similar D pocket and P3 conformation of B19/IL9 as in B19/RY8. G, The P5-His of peptide PA124 occupies the D pocket of B15/PA124, with P3-Glu pointing out of the D pocket.

When we compared the conformation of the same peptide RY8 presented by BF2*1901 and BF2*1501, we found the P3-Arg had distinct conformations within the two structures. The P3-Arg protrudes the side chain out of the D pocket of B19/RY8 groove (Fig. 5A), while in the B15/RY8 structure, the P3-Arg anchors the side chain into the D pocket (Fig. 5B). Furthermore, we aligned available BF2*1901 and BF2*1501 structures, and found the P3 anchor of peptides presented by BF2*1501 can accommodate different conformations with the side chains pointing into or outside the D pocket. In contrast, the P3 anchors of peptides presented by BF2*1901 all protrude out of the D pocket (Fig. 5C). Further analysis found the shallow and narrow D pocket of B19/RY8 groove is occupied by the large positive charged residue Arg111 (Fig.5D, F), in contrast to the large D pocket of BF2*1501 with the small residue Ser111 (Fig. 5E, G). We calculated the solvent-accessible surface area of P3-Arg that is buried upon interface formation with the pocket D, and found P3-Arg of the peptide RY8 has a smaller buried area in BF2*1901 (146.93 Å^2^ for molecule 1 and 134.65 Å^2^ for molecular 2 in the asymmetric unit cell) than the one in BF2*1501 structure (168.51 Å^2^). In the structures of B19/RY8 and B19/IL9, P3-Arg (Fig. 5D) and P3-His (Fig. 5F) protrude their side chains out of the D pockets. Meanwhile, a π-π interaction can be observed between P3-His of peptide IL9 and the residue Tyr156 of BF2*1901 (Fig. 5F), which may partly compensate the weak binding of P3 side chain out of the BF2*1901 groove. In the structure of B15/RY8, the P3-Arg locates in the larger D pocket and the hydrogen bond between P3-Arg and Ser111 of BF2*1501 can be observed. In contrast, the P3-Glu points out of the D pocket in the groove of B15/PA124 due to the presence in the D pocket of P5-His of peptide PA124. These analyses indicate a flexible but tight binding of P3 anchor of BF2*1501 compared to BF2*1901.

### The higher middle portion of α2 helix of BF2*1901/RY8 for TCR docking

**Fig 6:**
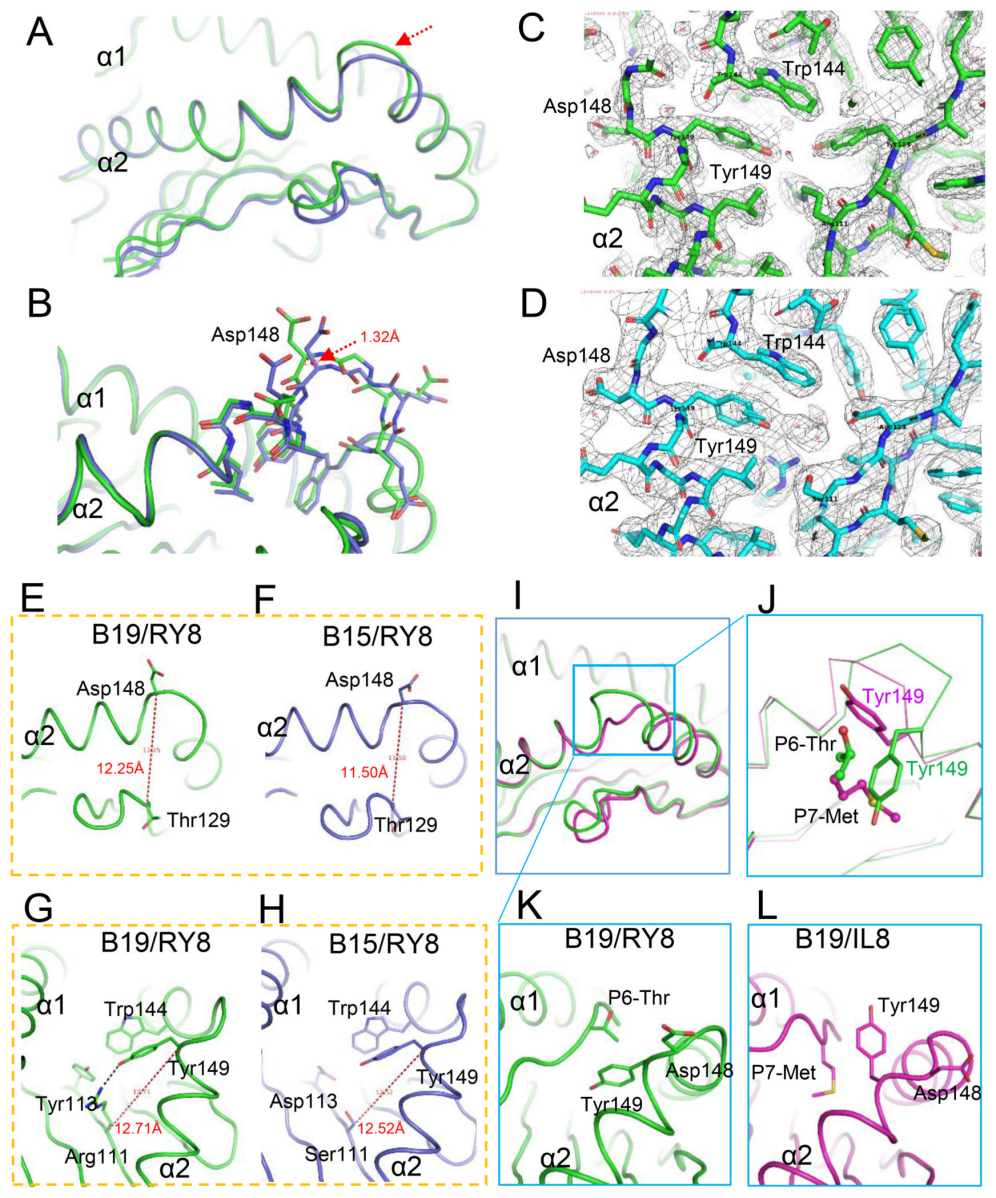
The higher α2 helix of BF2*1901/RY8 compared to BF2*1501/RY8 and BF2*1901/IL9. A, Superimposed structures of B19/RY8 (green) and B15/RY8 (PDB: 6LHH, blue), according to the Cα of α1α2 domains (residues 1-180). The different conformations of the middle portion in the α2 helices are pointed by the red arrow. B, The atomic positions of related residues of B19/RY8 (green) and B15/RY8 (blue) including the backbone atoms showed with sticks. The distance between Cα of Asp148 in the two structures was shown in red. C and D, The composite OMIT maps of α1 helices from B19/RY8 (green) and B15/RY8 (cyan). E and F, The distance measurement between the residues in the middle portion of α2 helix and the β sheets in B19/RY8 and B15/RY8. The distance between the Cα atoms of Asp148 and Thr129 in B19 (E) is longer than in B15 (F) shown in red dashed line. G and H, The two larger residues Arg111 and Tyr113 jack up the α2 helix of BF2*1901 through the interaction with Tyr149 and Trp144. The distance between the Cα atoms of Asp149 and Arg111 in B19 (G) is longer than the one between Asp149 and Ser111 in B15 (H). The hydrogen bond between Tyr149 and Arg111 is shown in black dashed line. I, Superimposed B19/RY8 (green) and B19/IL9 (purple) according to the Cα of α1α2 domains. The different conformations of the middle potion in the α2 helices were shown in the blue square. J, In the structural comparison between B19/RY8 (green) and B19/IL9 (purple), the large residue P7-Met of peptide IL9 pushes Tyr149 out of the peptide binding groove, which is different for P6-Thr of peptide RY8. K and L, The detailed conformational comparison between of B19/RY8 and B19/IL9.

In addition to the detailed analysis of the peptide anchoring of BF2*1901 and BF2*1501, we analyzed the MHC heavy chain itself in these two closely-related chicken MHC I molecules. The superimposition of B19/RY8 and B15/RY8, according to the Cα of α1/α2 domains (residues 1-180) showed different conformations of the middle potion in the α2 helices of the two structures (Fig. 6A). The middle portion of α2 helix of BF2*1901, covering Trp144 to Tyr149 had a higher position compared to the corresponding residues of BF2*1501. The distance between the Cα atoms of Asp148 of BF2*1901 and BF2*1501 is 1.32 Å (Fig. 6B). The composite OMIT maps of α1 helices from B19/RY8 and B15/RY8 showed the reliable atomic positions (Fig. 6C, D). The structure analysis showed that the two larger residues Arg111 and Tyr113 from the β-sheet of BF2*1901 jack up the α2 helix through the interaction with Tyr149 and Trp144 of α2 helix (Fig. 6E, G). In contrast, the α2 helix of B15 touches down due to the short sidechains of residues Ser111 and Asp113 in the β sheet (Fig. 6F, H).

The distance measurement between the residues in the middle portion of α2 helix and the β sheets also confirmed the higher position of α2 helix of B19 compared to BF2*1501. Asp148, as the highest residue on the α2 helix, has a longer distance to Thr129 from the β sheet in BF2*1901 (12.25 Å between the Cα of two residues) than in BF2*1501 (11.50 Å between the Cα of two residues) (Fig. 6E, F). The distance between the Cα atoms of Tyr149 and Arg111 in BF2*1901 (12.71 Å) is longer than in BF2*1501 (12.52 Å) (Fig. 6G, H).

Interestingly, in the two structures of BF2*1901 we determined here, the middle potion in the α2 helix of B19/IL9 shows a distinct conformation compared to the corresponding position of B19/RY8 (Fig. 6I). The superposing of the two structures showed that the different secondary anchor residue of peptides RY8 and IL9 lead to the conformation shift of the α2 helices in the two structures. The large residue P7-Met of peptide IL9 pushes the Tyr149 out of the peptide binding groove, which is different for the residue P6-Thr of peptide RY8 (Fig. 6J-L). The middle portion in the α2 helix locates at the highest position in a so-called super-bulged conformation of the TCR docking surface (62, 63). The conformational specificity of BF2*1901 at this region may lead to uncommon TCR docking strategy, which may imply a limited TCR repertoire for BF2*1901.

### The binding capacities of BF2*1901 and BF2*1501 to peptides

**Fig 7:**
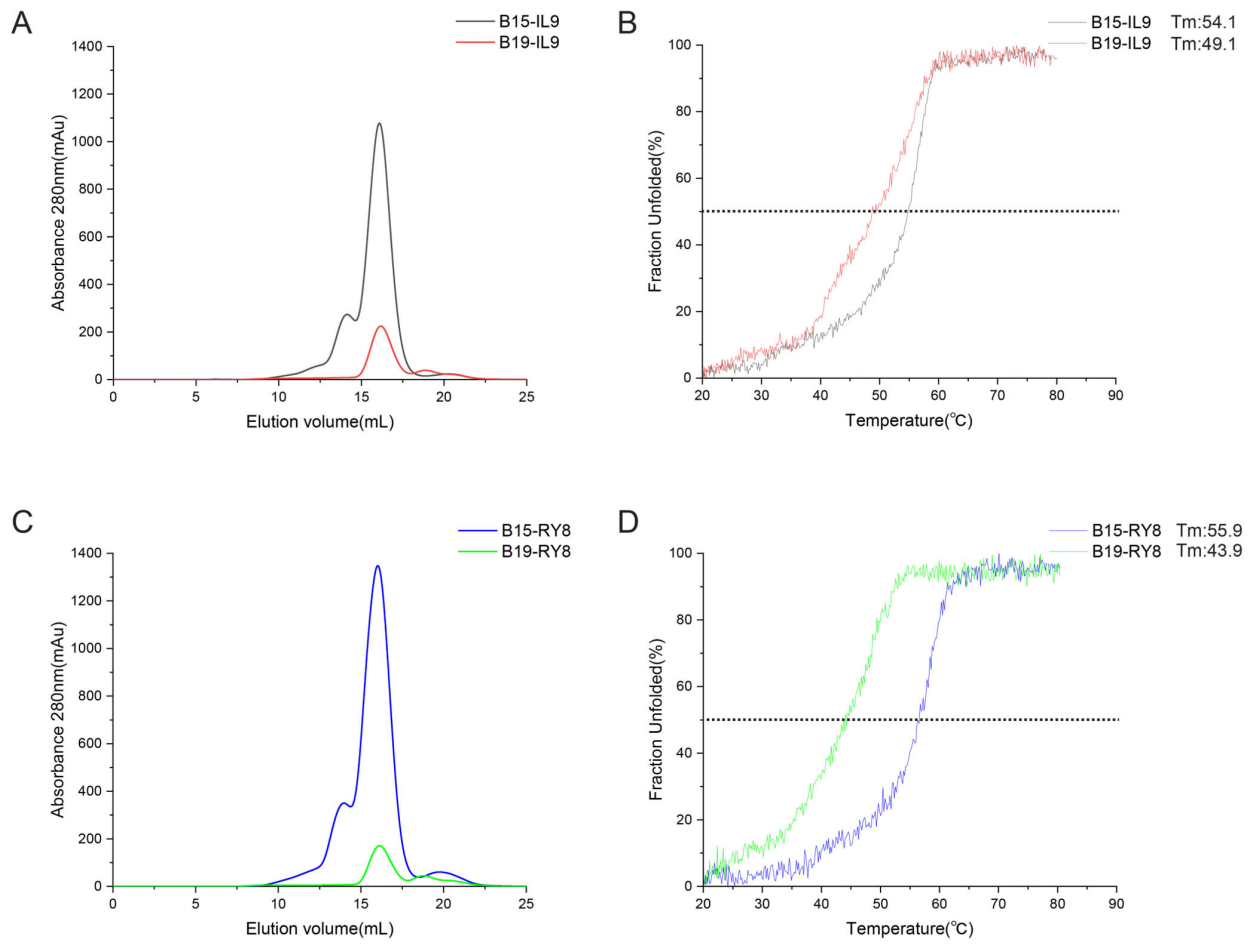
The weaker binding capacity of BF2*1901 compared to BF2*1501 with similar peptides. A and C, binding of peptides (A, IL9 and C, RY8) to BF2*1501 and BF2*1901 by *in vitro* refolding. The absorbance peak of the BF2 complex with the expected molecular mass of 45 kDa was eluted at the estimated volume of 16 mL on a Superdex 200 10/300 G L column. B and D, Thermostability of peptides (B, IL9 and D, RY8) complexed to BF2*1501 and BF2*1901 by CD spectroscopy showed by the curves generated from the raw data. The *T*_mS_ of different complexes are indicated by the temperature when 50% of the protein unfolded at the black dashed line. The experiments were independently performed twice.

To compare the binding capacities of BF2*1901 and BF2*1501 with a similar peptide, we utilized the peptides IL9 and RY8 to facilitate the *in vitro* renaturation of the two BF2 alleles followed by size exclusion chromatography (gel filtration) analyses. For the binding to either IL9 or RY8, BF2*1901 generated relatively lower yields of refolded products compared to BF2*1501 at the size expected for a class I monomer (Fig. 7A, C). The binding stabilities of the peptides IL9 or RY8 with BF2*1901 and BF2*1501 were further analyzed by CD spectroscopy (Fig. 7B, D), with the *T*ms determined from melting curves. BF2*1501 complexed with peptides IL9 or RY8 were more stable, with *T*ms of 54.1°C and 55.9°C, respectively. In contrast, BF2*1901 bound to IL9 or RY8 displayed significantly decreased stability with lower *T*ms of 49.1°C and 43.9°C, respectively, consistent with the narrower and shallower groove.

## Discussion

The correlation of resistance to Marek’s disease with the size of peptide repertoires for chicken class I (BF2) molecules is very clear (15, 18, 19, 22, 26), but the reasons why the B19 haplotype confers more susceptibility than the B15 haplotype, why the cell surface class I level of B19 cells is higher than B15 cells, and whether the BF2*1901 molecule has more fastidious peptide-binding than BF2*1501 have all remained unclear. In this paper, we confirm that the viral loads after MDV infection of B19 chickens are much higher than of B15 chickens, describe and compare the detailed peptide motifs from B19 cells versus B15 cells, and show by multiple crystal structures how the narrow and shallow peptide-binding groove of BF2*1901 molecules can result in a less promiscuous binding than the relatively larger and deeper groove of BF2*1501.

We have recently derived chicken lines with various MHC haplotypes and examined some of them (including the line bearing the B19 haplotype) for response to MDV (43). Here we use RT-qPCR to show that the basal levels of various cytokines are generally similar in the lines with B15 and B19, but with higher active cytokine IFN-γ and lower inhibitory cytokine IL-10 in B15 chickens. Furthermore, the virus levels determined by qPCR after MDV infection begin to differ sharply at 12 dpi. Much more virus is found in B19 chickens, in agreement with the published hierarchy of susceptibility to Marek’s disease (12).

We also present detailed evidence for the self-peptides bound to class I molecules presented by B19 cells: sequences from individual peptides and peptide pools from blood and spleen cells by gas phase sequencing, as well as peptides with Arg at P2 from an MDV-transformed cell line by immunopeptidomics. These results confirm and extend the B19 class I motif originally described (21), but they fail to explain in any obvious way the relative MDV susceptibility of B19 compared to B15 chickens in terms of peptide repertoire. In comparison with the sequences of individual peptides and peptide pools from peptides of B15 cells presented previously (15, 21), both molecules require an Arg at P2, but B15 prefers a basic residue at P1 and a Tyr at Pc compared to multiple amino acids found for B19 at both positions. Thus, the dominantly-expressed class I molecule of B19 might seem more promiscuous based on the peptide motifs than the class I molecule of B15, which is the opposite of what has been seen up to now in terms of MDV resistance (18, 19).

We resolve this conundrum using structures of B15 and B19 class I molecules (BF2*1501 and BF2*1901) bound to multiple peptides, including the same peptide (RY8) bound to both molecules. Although both B15 and B19 molecules bind the amino terminus of the peptide in pocket A and require Arg as the anchor residue at P2 in a deep pocket B containing Asp24, the B19 molecule has many larger residues leading to an overall narrower and shallower peptide-binding groove in pockets C, D, E and F. The larger Trp95 and Tyr113 of BF2*1901 leads to a much narrower and shallower pocket F than Leu95 and Asp113 of BF2*1501. Thus, the various amino acids found at the C-terminal anchor residue of BF2*1901 are likely bound with much less affinity (with therefore likely fewer total peptides) than the Tyr overwhelmingly favored by BF2*1501. Similarly, the larger Arg111 residue of BF2*1901 leads to a much narrower and shallower pocket D than Ser111 of BF2*1501. The side chains of residues at P3 are all forced out of the groove in BF2*1901, whereas most are accommodated (as so-called secondary anchors) in larger pocket D of BF2*1501, with one exception due to the peptide residue at P5 occupying pocket D. Thus, the location of the middle of the peptide is higher out of the groove, with again likely less affinity and fewer numbers of peptides for BF2*1901. Also, Trp95 in BF2*1901 is much larger than Leu95 in BF2*1501, so that pocket C is similarly affected, as shown by the deeper anchoring of P5-Asn of peptide RY8 in BF2*1501 compared to P5-Asn of peptide RY8 in BF2*1901 (Fig. 3G,H). Thus, the wider and deeper peptide-binding groove of BF2*1501 means that more peptides can be bound, as opposed to BF2*1901 for which only fewer peptides with the highest affinity will bind. Meanwhile, our BF2*1901 structure showed a π-π interaction between P3-His of peptide IL9 and the residue Tyr156 of BF2*1901 (Fig. 5F), which partly compensates the weak binding of P3 anchor out of the BF2*1901 groove. This may explain the why >50% BF2*1901-binding peptides prefer Phe or Tyr as P3 anchor based on the immunopeptidomic data.

The finding that BF2*1901 has a narrower and shallower PBG than BF2*1501 was unexpected, and the argument that these properties lead to a narrower range of peptides bound but at a higher affinity *in vivo* is subtle and could be considered counter-intuitive. The fact that the class I molecules are more thermostable from B19 compared to B15 blood and spleen cells (23) may reflect BF2*1901 at the cell surface bearing only those peptides with the highest affinity (whatever the sequence), whereas BF2*1501 can accommodate a greater variety of peptides in the wider and deeper PBG (thus including a wider range of affinities). In this view, the fact that the predicted peptide RY8 bound less strongly to BF2*1901 than to BF2*1501 would mean that it is unlikely that this peptide would be found at the surface of B19 cells.

Also potentially relevant are the peptides available for binding *in vivo*, which depend on the evolutionary history of the B15 and B19 haplotypes. Many chicken MHC haplotypes (including B15) appear to be very stable in evolution, with the peptide-translocation specificity of B15 TAP molecules extremely similar to the peptide-binding specificity of BF2*1501 (23, 28). The peptide reservoir from the conserved protein regions of virus may contribute to the protective immune response and memory (64, 65). In contrast, B19 is clearly a recombinant haplotype, with TAP genes derived from the B12 haplotype and thus evolved to pump peptides for the much more promiscuous BF2*1201 (which has a completely different peptide motif than B15 and B19) (15, 28), perhaps leading to a wider variety of peptides available in B19 cells, of which far fewer would be appropriate for BF2*1901.

Despite both molecules having Glu65 which could bind to basic residues at peptide position P1 (as in the human class I molecule HLA-B27 (61), the conformation of the amino acids at P1 of peptides varied considerably, even for the peptides bound to BF2*1501 which all have basic amino acids at P1. For only the one peptide PA124, the basic sidechain of P1-Arg bound to BF2*1501 by a salt bridge (Fig. 4C) with Glu65 (2.90 Å) and a hydrogen bond with Tyr61 (2.89 Å). Although in molecule 1 of BF2*1901/RY8 asymmetric unit, sidechain of P1-Arg from peptide RY8 bound to Glu65 of BF2*1901 with a weak hydrogen bond (3.58 Å), no interaction of P1-Arg with residue of BF2*1901 can be observed in molecule 2 of BF2*1901/RY8. The reason for these differences remains mysterious, but it still indicates a tight anchoring of P1 residue of one BF2*1501-binding peptide compared to the ones of BF2*1901.

Finally, the larger residues Arg111 and Tyr113 from the β-sheet interact with Tyr149 and Trp144 of the α2 helix to raise the middle of the α2 helix in BF2*1901, compared to BF2*1501 which has Ser111 and Asp113. However, in previously determined MHC class I structures, peptides have been shown to alter the conformation of side chains and even the backbone of the helix in peptide-dependent ways (66, 67). The conformational changes of the helix in the BF2*1901/RY8 structure are consistent with this possibility. In any case, such conformational changes of the BF2*1901 α2 helix, whether peptide-dependent or not, may lead to a super-bulged surface affecting the binding to TCRs (62, 63). Previously it has been speculated that the peptide repertoire of MHC molecules may affect the T cell repertoire (18, 19), but this observation of a fastidious class I molecule that may not be easily recognized by most TCRs provides a new mechanism by which this situation might occur.

In summary, we provide further evidence that B19 chickens are more susceptible to MDV than B15 chickens, we conduct the first detailed analysis of self-peptides leading to the peptide motif of BF2*1901, we present two structures of BF2*1901, and we compare several structures of BF2*1501 and BF2*1901. We find that the self-peptides bound to BF2*1901 may appear more various than those of BF2*1501, but that the structures show the narrower and shallower peptide-binding groove of BF2*1901 means that it will accept fewer peptides overall, with those present *in vivo* likely having the highest affinity. This finding is consistent with the width and depth of the whole range of promiscuous to fastidious molecules (18, 22, 25, 26), and suggests that the peptides found bound to BF2*1901 are the very best binders to a narrow and shallow groove, in which many parts of the peptide binding are important, not just the particular amino acids in the positions of the anchor residues. Our data confirm that viremia for MDV is higher in the B19 than the B15 chicken lines, showing a different susceptibility to Marek’s disease. The structures explain the greater promiscuity for the BF2 molecules from B15 compared to B19, correlating with the facts that B19 class I molecules have higher surface expression and greater stability *in vivo* (23).

## Supplementary Material

1

## Figures and Tables

**Table 1:** X-ray data processing and refinement statistics.

Parameter	BF2*1901/RY8	BF2*1901/IL9
PDB code	7WBG	7WBI
Data collection statistics		
Space group	P2_1_2_1_2_1_	P2_1_2_1_2_1_
Cell parameters (Å)		
a (Å)	78.13	47.96
b (Å)	85.03	76.15
c (Å)	110.84	102.91
α (°)	90.00	90.00
β (°)	90.00	90.00
γ (°)	90.00	90.00
Wavelength (Å)	0.97853	1.54178
Resolution (Å)	50.0-2.0(2.07-2.0)^a^	50.0-1.80 (1.86-1.80)
Total reflections	377910	386451
Completeness (%)	98.2(99.9)	99.9(100.0)
Redundancy	6.3(7.6)	10.7(10.8)
R_merge_(%)^b^	6.8(14.2)	4.5(20.7)
I/σ	22.5(13.1)	53.9 (11.7)
Refinement statistics		
R_work_(%)^c^	18.7	16.6
R_free_(%)	23.6	19.5
RMSD		
Bonds (Å)	0.008	0.01
Angle (°)	1.23	1.19
Average B factor	27.36	19.73
(Å^2^)		
Ramachandran plot quality (%)		
Favored (%)	99.17	98.65
Allowed (%)	0.83	1.35
Outliers (%)	0	0

a Numbers in parentheses represent the highest-resolution shell.

b R_merge_ = ∑_hkl_∑_i_|I_i_−<I>| ∑hkl∑_i_ I_i_, where I_i_ refers to the observed intensity and <I> is the average intensity of multiple observations of symmetry related reflections.

c R =∑_hkl_||F_obs_|−k|F_call_|| /∑_hkl_|F_obs_|, where R_free_ is calculated for a randomly chosen 5% of reflections and R_work_ is calculated for the remaining 95% of reflections used for structure refinement.

## Key Points

- Chicken haplotype B19 confers greater susceptibility to Marek’s disease than B15.
- BF2*1901 binds peptides with a greater variety of specific anchors than BF2*1501.
- Narrower and shallower groove of BF2*1901 confers weaker binding to most peptides.
