# Supplementary material for: A wider and deeper peptide-binding groove for the class I molecules from B15 compared to B19 chickens correlates with relative resistance to Marek’s disease
Source: J Immunol. Author manuscript; Available in PMC 2023 Mar 9. (PMC7614295; doi:10.4049/jimmunol.2200211)
Supplement: 1 [file EMS159211-supplement-1.pdf]

SUPPLEMENTARY INFORMATION

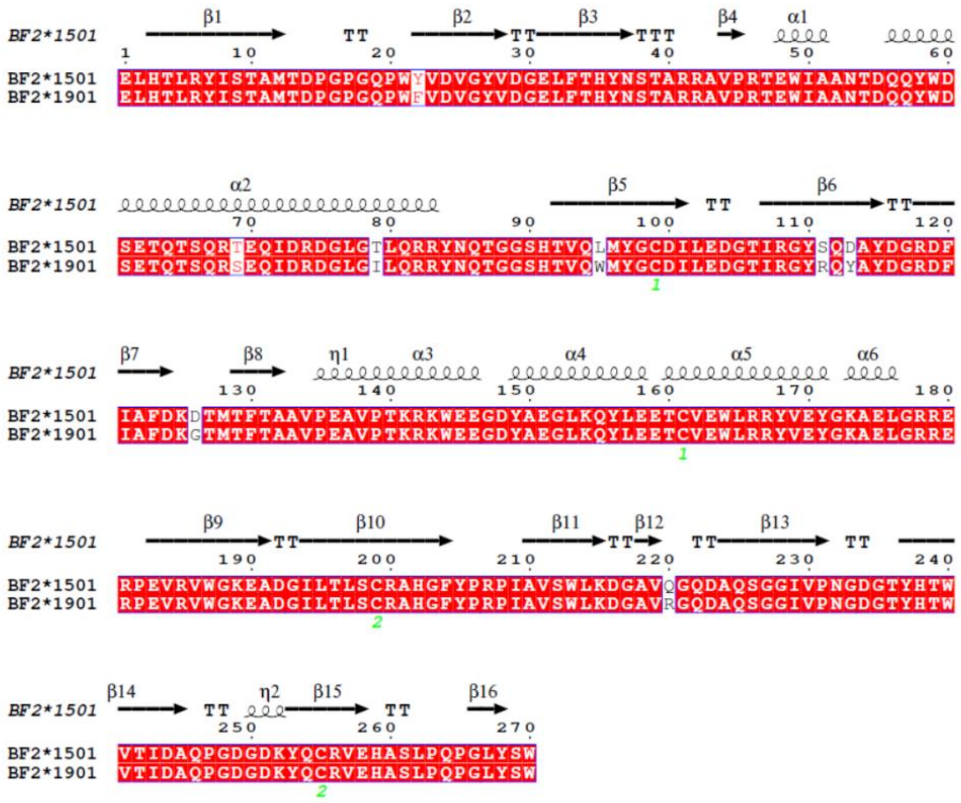

Fig. S1 Structure-based sequence alignment of BF2*1901 and BF2*1501.

Cylinders indicate α-helices, and black arrows indicate β-strands. Residues highlighted in red are completely conserved, and residues in blue boxes are highly (>80%) conserved. Residues that play a critical role in the conformations of Mamu-A*02-presented peptides are marked with deep blue asterisks. The sequence alignment was generated with Clustal X and ESPript.

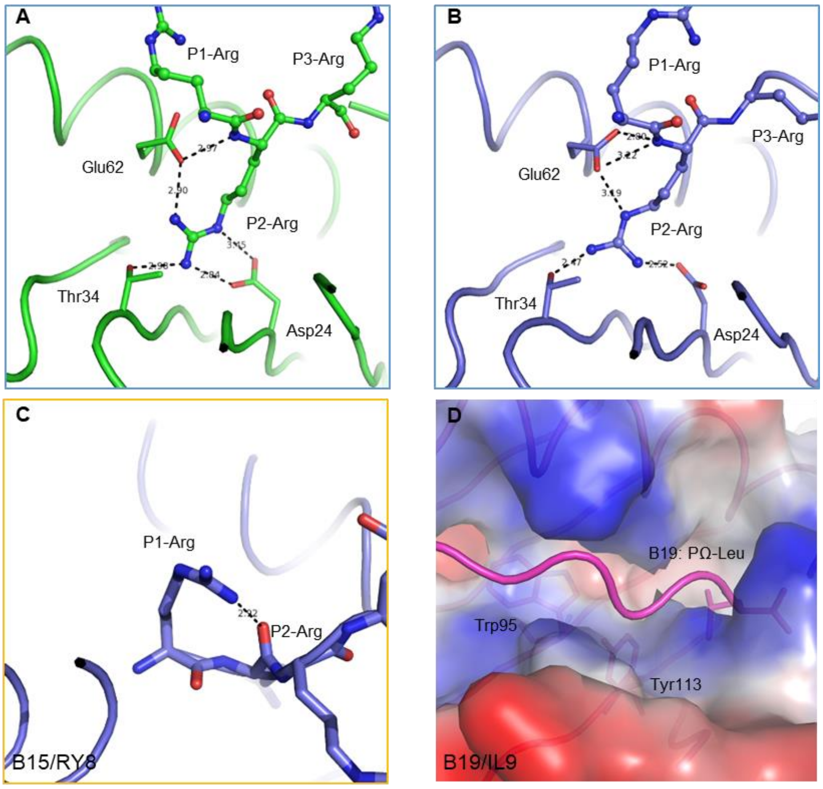

Fig. S2 The detailed comparison of BF2*1501 and BF2*1901.

A, Structure of B pocket in B19/RY8 (green). B, B pocket of B15/RY8 (PDB: 6LHH, blue). The hydrogen bond between P2-Arg of peptide RY8 in B19/RY8 and B15/RY8 are shown in black dashed lines. C, The intra chain hydrogen bond of P1-Arg in the B15/RY8 (Blue). D, The PΩ-Leu of peptide IL9 in BF2*1901/IL9 structure inserts its side chain into Pocket F of BF2*1901. The electrostatic plot shows the narrow and shallow F pocket of B19 with peptide IL9 in purple sticks.

Table S1. Peptides used for the renature and crystallization of BF2*1501 and BF2*1901.

Name	Sequence	Pathogens	Protein	Position
**RY8**(B15-2)	R**R**REQTD**Y**	Marek's disease virus	MEQ	74-81
**IL9**(B19-1)	I**R**HENRMV**L**	H1N1,H3N2,H5N1,H7N9,H9N2	M1	282-290
PY9(B19-2)	P**K**KTGGPI**Y**	H1N1,H3N2,H5N1,H7N9,H9N2	NP	89–97
KF9(B19-3)	K**R**GINDRN**F**	H1N1,H3N2,H5N1,H7N9,H9N2	NP	204–212
LF9(B19-4)	L**K**PSDTIN**F**	H5N8	HA	249-257
